# Clinical characteristics and factors associated with recurrence and long-term prognosis in patients with MOGAD

**DOI:** 10.3389/fimmu.2025.1535571

**Published:** 2025-05-08

**Authors:** Wenlin Sun, Yinyin Xie, Aoya Han, Xinru Zhou, Shijie Zhang, Yi Xie, Nanchang Xie

**Affiliations:** Department of Neurology, The First Affiliated Hospital of Zhengzhou University, Zhengzhou, China

**Keywords:** MOGAD, Myelin oligodendrocyte antibody, clinical phenotypes, prognosis, retrospective cohort study

## Abstract

**Objective:**

To describe the clinical features, treatment, and prognostic factors affecting recurrence and long-term adverse outcomes of myelin oligodendrocyte antibody-associated disease (MOGAD).

**Methods:**

In this retrospective cohort study, the records of patients diagnosed with MOGAD at Zhengzhou University First Affiliated Hospital between January 2018 and March 2023 were analyzed, and factors associated with recurrence and poor long-term prognosis were identified using logistic regression.

**Results:**

Of the 91 patients, 69 (76%) were new cases, 39 (43%) were female, and 47 (52%) were children (<18 years). Clinical manifestations included optic neuritis (ON) in 13 (14%), transverse myelitis (TM) in 14 (15%), brain disease in 37 (41%), and mixed encephalomyelitis in 27 (30%). The prevalence of acute disseminated encephalomyelitis (ADEM) was significantly higher in children than in adults (43% versus 18%, p = 0.012), whereas the prevalence of TM was significantly higher in adults (30% versus 2%, p < 0.001). Combined steroid and intravenous immunoglobulin (IVIG) treatment during hospitalization was more frequent in children than in adults (36% versus 11%, p = 0.006), and children had a better short-term prognosis than that in adults at discharge (median [interquartile range (IQR)]) Expanded Disability Status Scale [EDSS]: 1 [0–1] versus 2 [0–4.75], p = 0.007; Modified Rankin Score [mRS]: 1 [0–1] versus 1 [0–2], p = 0.006). Visual impairment was a risk factor for recurrence (odds ratio [OR]: 4.22, 95% confidence interval [CI]: 1.24–14.38, p = 0.022). A higher EDSS score at discharge (OR: 5.05, 95% CI: 1.27–20.07, p = 0.021)and more previous episodes (OR: 9.24, 95% CI: 1.35–63.10, p = 0.023), were associated with a poor long-term prognosis; whereas steroid therapy for >5 weeks at first diagnosis (OR: 0.001, 95% CI: 0.00–0.33, p = 0.019) and type I isoelectric focusing pattern (OR: 0.004, 95% CI: 0.00–0.402, p = 0.043) were associated with favorable long-term prognosis.

**Conclusion:**

After the first episode, steroid maintenance for an appropriate period following discharge is important for achieving a favorable long-term prognosis, particularly in patients with a high EDSS score at discharge and those at a heightened risk of recurrence.

## 1 Introduction

Myelin oligodendrocyte glycoprotein (MOG), a protein localized to the outermost lamella of the myelin sheath that serves as a key developmental marker for oligodendrocyte maturation, plays critical roles in both maintaining myelin sheath integrity and regulating cellular communication processes within the human central nervous system (1, 2). In the 1980s, MOG was identified as a target in experimental autoimmune encephalomyelitis (3, 4), however, early ELISA assays showed that MOG antibodies were widely present in both healthy individuals and multiple sclerosis patients, lacking specificity (5, 6). With advancements in antibody detection methods, specific MOG-IgG has been detected in non-multiple sclerosis (MS) patients with conditions such as optic neuritis and acute disseminated encephalomyelitis (7). Live cell-based assays further confirmed that 30%-70% of seronegative neuromyelitis optica spectrum disorders (NMOSD) patients carry MOG-IgG (8–10). It is currently believed that MOG antibody-associated disease (MOGAD) is an acquired demyelinating disease of the central nervous system, distinct from MS and NMOSD, and the affected patients may exhibit any combination of acute disseminated encephalomyelitis (ADEM), transverse myelitis (TM), optic neuritis (ON), brainstem syndrome (BS), and cortical encephalitis (CE) (11). Although the International MOGAD Panel established diagnostic criteria for the disease in 2023 (12), formal treatment guidelines remain unavailable, with current therapeutic recommendations primarily based on empirical protocols (13). Previous studies indicate that MRI lesions following acute MOGAD attacks tend to resolve completely, which significantly differs from AQP4-IgG+ NMOS and MS (14, 15). However, 11%-47% of patients may experience relapses within 2 years (16), with the recurrence risk increasing to 72% as follow-up extends (17). Additionally, up to 45% of patients may develop permanent disability (18–20). Several aspects of MOGAD, such as phenotypic heterogeneity and disease trajectory need to be characterized, and risk factors associated with recurrence and poor long-term prognosis need to be identified to guide individualized treatment.

The purpose of this study was to describe clinical manifestations, diagnostic investigations, and treatment of MOGAD, and identify risk factors associated with recurrence and poor long-term prognosis.

## 2 Methods

### 2.1 Participants

Patients treated for MOGAD at the First Affiliated Hospital of Zhengzhou University in Zhengzhou, Henan, China between January 2018 and March 2023 were included in a retrospective cohort study. The inclusion criteria were: (i) meeting the diagnostic criteria for MOGAD proposed by Banwell et al. (12); (ii) having at least one clinical episode lasting more than 24 hours within the past month; and (iii) follow up for at least 6 months. The exclusion criteria were: (i) missing key clinical data or laboratory test results; or (ii) the presence of other autoimmune encephalitis and/or demyelinating antibodies in addition to myelin oligodendrocyte (MOG) antibodies.

Patients were divided into child (age <18 years) and adult (age ≥18 years) groups. The cohort was also divided into four clinical phenotypes: isolated ON, isolated TM, brain (isolated CE, isolated ADEM, isolated BS, or other cerebral syndromes), and mixed groups, according to the clinical manifestations. Patients who visited the hospital for their first episode and were diagnosed with MOGAD were defined as the incident cohort. The incident cohort was divided into single episode and recurrent groups according to whether patients experienced a recurrence (new neurological symptoms or signs lasting > 24 hours, occurring more than one month after the previous episode) during the follow-up period. The incident cohort was also divided into good and poor prognosis groups according to their Expanded Disability Status Scale (EDSS) score (< 2 vs. ≥ 2) and Modified Rankin Score (mRS) (≤ 2 vs. > 2) at the most recent follow up.

The study was approved by the Ethics Committee of the First Affiliated Hospital of Zhengzhou University (2024-KY-0810), which waived the requirement for written informed consent owing to the retrospective study design.

### 2.2 Clinical data

Data were collected on: (i) Demographic and clinical characteristics: age at onset, sex, triggers and prodromal manifestations, clinical manifestations, number of episodes, and disease course. (ii) Laboratory test results: Cerebrospinal fluid (CSF) cell count, glucose, protein, albumin (Alb), and immunoglobulin G (IgG) levels; Alb quotient (QAlb); IgG quotient (QIgG); IgG index; intrathecal IgG synthesis rate; and isoelectric focusing pattern (21); damage to the blood-brain barrier (BBB) (elevated CSF Alb/serum Alb ratio greater than, with the normal value calculated as = (4 + age/15) × 10^-3^) (22). Virus antibodies detection in CSF and serum included Epstein-Barr virus, cytomegalovirus, coxsackie virus, measles virus, herpes simplex virus, human parvovirus B-19, influenza b virus, parainfluenza virus, adenovirus, rubella virus, herpes zoster virus, and echovirus. MOG-IgG testing methodology: CSF and/or serum samples were collected pre-treatment from patients and analyzed at either the Neurology Department Laboratory of the First Affiliated Hospital of Zhengzhou University or Zhengzhou Golden System Clinical Laboratory Center. Both facilities employed live cell-based assays (CBA) using fluorescein isothiocyanate (FITC)-conjugated antibodies, with fluorescence microscopy evaluation. The positivity criteria differed between institutions: At Zhengzhou University Hospital, samples were deemed positive based on distinct fluorescence signals (subjective visual assessment); the Golden Center utilized a semi-quantitative approach, determining positivity through fluorescence signal analysis followed by titer calculation relative to standardized quality control substances. (iii) Imaging and electrophysiological investigation results: magnetic resonance imaging (MRI) of the head, spinal cord, and optic nerve; electroencephalogram (EEG); visual evoked potential (VEP); electroretinogram (ERG); optical coherence tomography (OCT); and fundus photography. (iv) Treatment: Immunotherapy in the acute phase included intravenous steroids, intravenous immunoglobulin (IVIG), and plasma exchange. Maintenance immunotherapy included low-dose steroids, mycophenolate mofetil, azathioprine, methotrexate, tacrolimus, rituximab, and ofatumumab. (v) Disease severity and prognosis: intensive care unit (ICU) admission; mRS and EDSS on admission, discharge, and most recent follow up. All patients were followed up by telephone or outpatient consultations.

### 2.3 Statistical analysis

Data analysis was performed using SPSS v25.0 (IBM Corp., Armonk, NY, USA). Medians with ranges or interquartile ranges (IQRs), or means and standard deviations, were used to describe continuous variables. Qualitative variables were expressed as frequencies and percentages, or constituent ratios. Independent-samples t-tests and Pearson correlations, or Mann-Whitney U-tests and Spearman correlations, were used to compare continuous variables. Chi-squared tests or Fisher’s exact test were used to compare categorical variables. Single variable and multivariable binary logistic regression were used to identify risk factors. Two-sided p values < 0.05 were considered statistically significant.

## 3 Results

### 3.1 Demographic characteristics and clinical presentation

**Figure 1:**
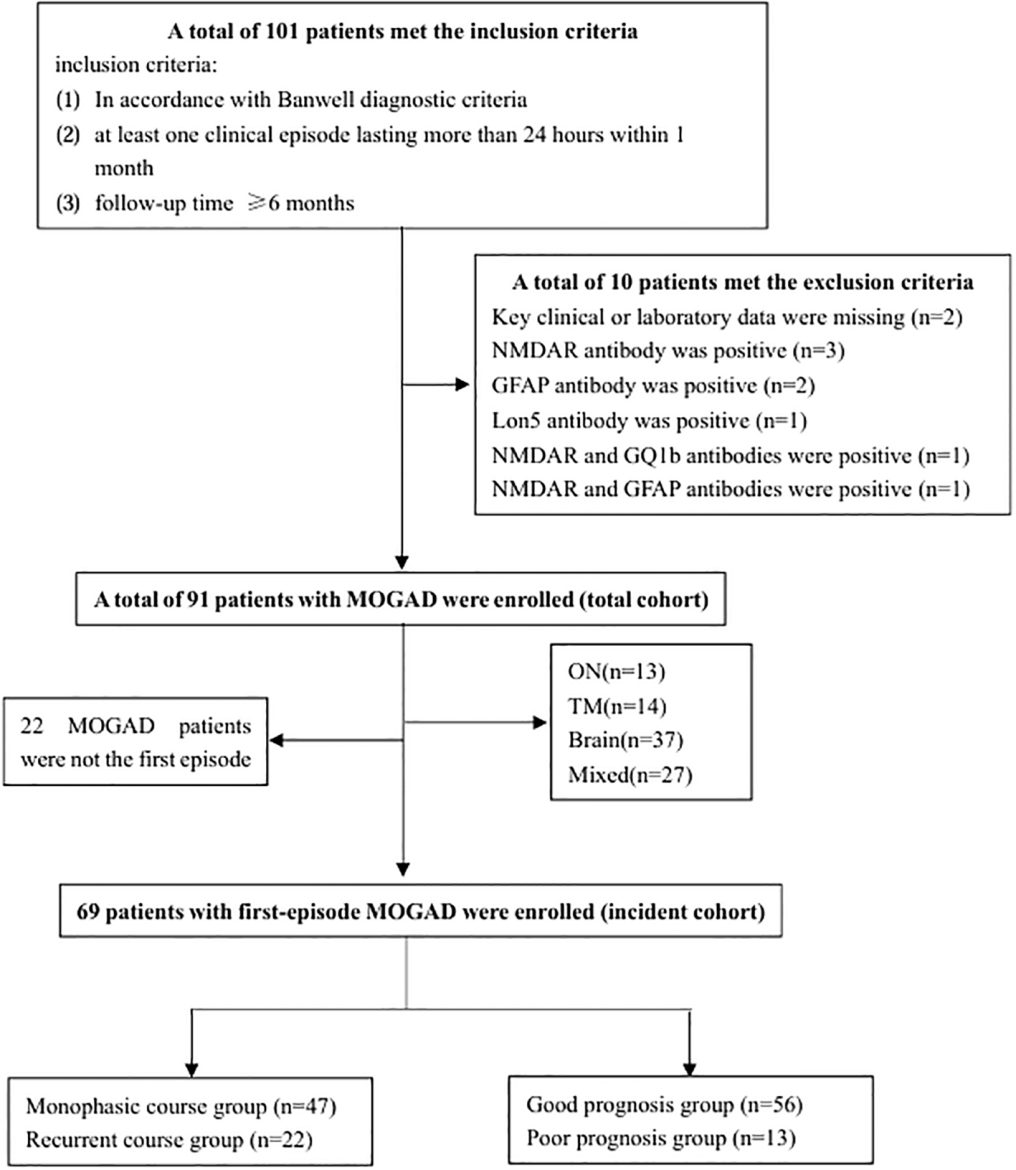
Flow chart of patient screening. GFAP, glial fibrillary acidic protein; MOGAD, myelin oligodendrocyte glycoprotein antibody-associated disease; NMDAR, N-methyl-D-aspartate receptor; ON, optic neuritis, TM, transverse myelitis.

**Figure 2:**
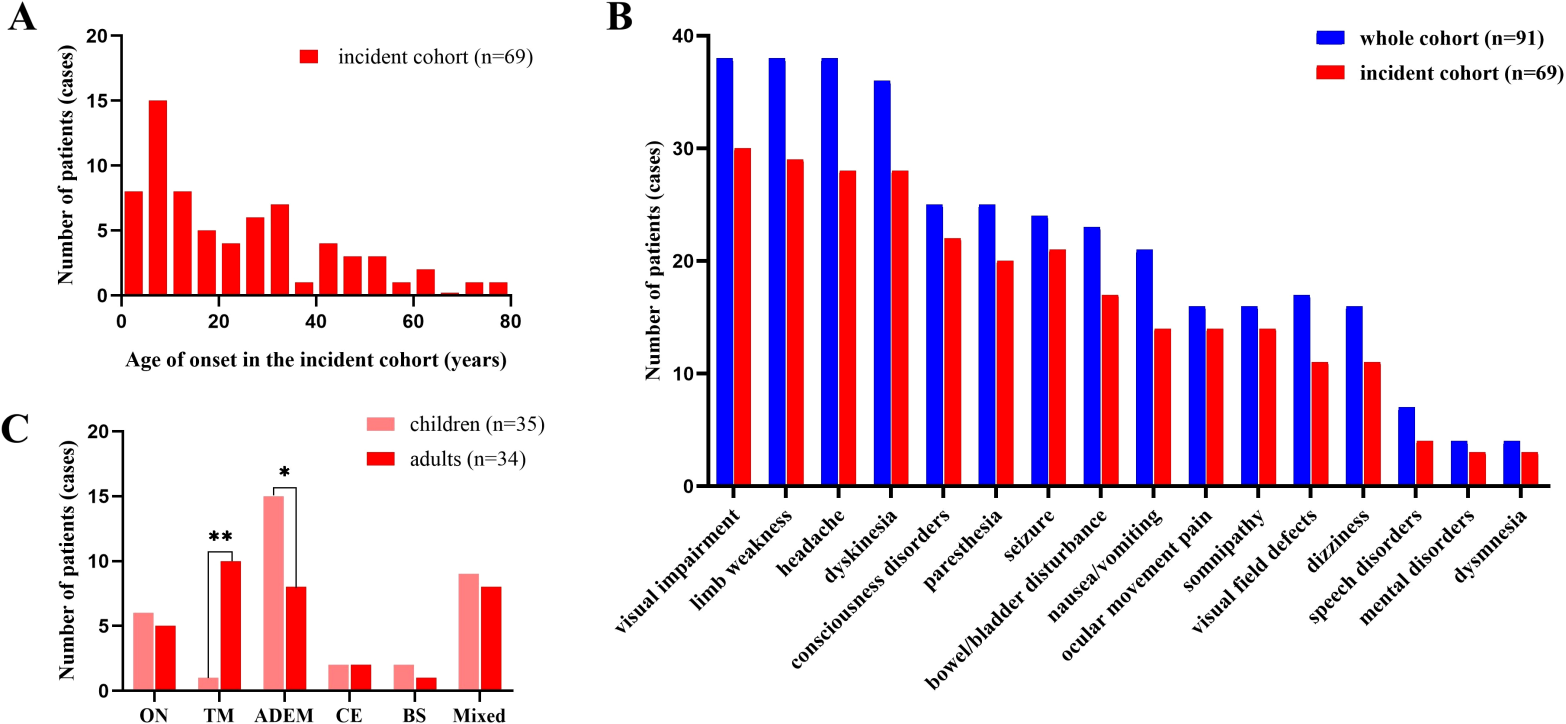
Demographic and clinical features of the cohort. **(A)** Age at disease onset in the incident cohort. **(B)** Clinical manifestations at onset in the whole cohort and incident cohort of patients with MOGAD. **(C)** Clinical phenotypes in children and adults in the incident cohort. Brain involvement was divided into three categories: CE, ADEM and BS. ADEM, acute disseminated encephalomyelitis; BS, brainstem syndrome; CE, cortical encephalitis; MOGAD, myelin oligodendrocyte glycoprotein antibody-associated disease; ON, optic neuritis, TM, transverse myelitis. *:p<0.05;**p<0.001.

A total of 91 patients were enrolled in the whole cohort (**Figure 1**). The age of onset ranged from 1 to 79 years, with a median of 17 years (interquartile range [IQR]: 8–34 years) and a peak age of onset between 5 and 10 years. Of the patients, 47 (52%) were children, 44 (48%) were adults, 39 (43%) were females, and 52 (57%) were males. Of the patients, 69 were in the incident cohort, with an age of onset ranging from 1 to 79 years, a median age of onset of 17 years (IQR: 7–34 years), and a peak age of onset between 5 and 10 years (**Figure 2A**). Of the patients in the incident cohort, 35 (51%) were children, 34 (49%) were adults, 28 (41%) were female, and 41 (59%) were males. Compared with the whole cohort, the incident cohort had a lower incidence of recurrence (32% versus 48%, p = 0.036).

The most common phenotype was ADEM (31%, 28/91), followed by the mixed type (30%, 27/91), TM (15%), and ON (14%), and isolated BS was the least common phenotype (4%, 4/91). A total of 193 episodes were observed throughout the disease course. ADEM was the most common type (43%, 83/193), followed by mixed type (23%), TM (13%) and ON (13.0%), and isolated BS (3%, 6/193) was the least common. In the whole cohort, visual impairment was the most common clinical manifestation (42%, 38/90), followed by headache (42%), limb weakness (42%), and dyskinesia (40%) (**Figure 2B**).

### 3.2 Laboratory, imaging, and electrophysiological investigations

The laboratory, imaging, and electrophysiological parameters in the whole cohort and incident cohort are shown in **Table 1**.

**Table 1:** Demographic and clinical characteristics of patients with MOGAD.

Variables	Total cohort (n=91)	Incident cohort (n=69)	P	Children (n=47)	Adults (n=44)	P
**Female, n (%)**	39/91 (42.9)	28/69 (40.6)	0.722	22/47 (46.8)	17/44 (38.6)	0.431
**Age of onset (years)**	17 (8,34)	17 (7,34)	1	8.49 ± 4.32	34 (26,47)	**<0.001**
Clinical symptoms						
**Fever, n (%)**	39/91 (42.9)	30/69 (43.5)	1	28/47 (59.6)	11/44 (25.0)	**0.001**
**Visual impairment, n (%)**	38/90 (42.2)	30/68 (44.1)	0.812	19/46 (41.3)	19/44 (43.2)	0.857
**Ocular movement pain, n (%)**	16/90 (17.8)	14/68 (20.6)	0.809	10/46 (21.7)	6/44 (13.6)	0.315
**Visual field defects, n (%)**	17/79 (21.5)	11/60 (18.3)	0.643	11/41 (26.8)	6/39 (15.4)	0.211
**Headache, n (%)**	38/91 (41.8)	28/69 (40.6)	1	20/47 (42.6)	18/44 (40.9)	1
**Dizziness, n (%)**	16/91 (17.6)	11/69 (15.9)	0.784	9/47 (19.1)	7/44 (15.9)	0.685
**Somnipathy, n (%)**	16/91 (17.6)	14/69 (20.3)	0.818	9/47 (19.2)	7/44 (15.1)	0.685
**Nausea/vomiting, n (%)**	21/91 (23.1)	14/69 (20.3)	0.673	19/47 (40.4)	2/44 (4.5)	**<0.001**
**Bowel/bladder disturbance, n (%)**	23/91 (25.3)	17/69 (24.6)	0.927	6/47 (12.8)	17/44 (38.6)	0.005
**Paresthesia, n (%)**	25/91 (27.5)	20/69 (29.0)	0.833	5/47 (10.6)	20/44 (45.5)	**<0.001**
**Limb weakness, n (%)**	38/91 (41.8)	29/69 (42.0)	1	17/47 (36.2)	21/44 (47.7)	0.264
**Seizure, n (%)**	24/91 (26.4)	21/69 (30.4)	0.571	17/47 (36.2)	7/44 (15.9)	**0.028**
**Dysmnesia, n (%)**	4/91 (4.4)	3/69 (4.3)	1	3/47 (6.4)	1/44 (2.3)	0.657
**Consciousness disorders, n (%)**	25/91 (27.5)	22/69 (31.9)	0.544	19/47 (40.4)	6/44 (13.6)	**0.04**
**Mental disorders, n (%)**	4/91 (4.4)	3/69 (4.3)	1	2/47 (4.3)	2/44 (4.5)	1
**Dyskinesia, n (%)**	36/91 (39.6)	28/69 (40.6)	0.896	16/47 (34.0)	20/44 (45.5)	0.266
**Speech disorders, n (%)**	7/91 (7.7)	4/69 (5.8)	0.878	6/47 (12.8)	1/44 (2.3)	0.138
**Admission to the ICU, n (%)**	33/91 (19.8)	15/69 (21.7)	0.762	15/47 (31.9)	3/44 (6.8)	**0.003**
Cerebrospinal fluid (CSF)						
**Cell count (×10^6/L)**	30.0 (5.0,61.5)	32.0 (7.75,76.0)	0.45	34.0 (14.0,79.5)	48.0 (14.0,79.2)	**0.031**
**Glucose (mmol/L)**	3.28 (2.78,3.60)	3.21 (2.77,3.69)	0.778	3.41 (2.80,3.95)	3.19 (2.78,3.51)	0.302
**Protein (mg/L)**	327.0 (257.0,419.0)	326.0 (259.4,423.0)	0.896	285.0 (223.5,385.0)	380.8 (307.8,562.6)	**0.001**
**Immunoglobulin IgG (mg/L)**	30.65 (20.70,44.05)	30.65 (23.80,45.20)	0.817	29.00 ± 2.94	36.60 (27.40,56.95)	**0.003**
**Albumin (mg/L)**	196.5 (143.9,259.4)	200.2 (145.7,278.5)	0.786	176.11 ± 12.28	236.9 (175.4,331.9)	**<0.001**
**QIgG,**	3.04 (2.14,4.51)	3.04 (2.17,4.46)	0.896	2.25 (1.92,3.84)	3.75 (2.62,5.68)	**0.003**
**QAlb**	4.55 (3.38,6.07)	4.60 (3.40,6.84)	0.719	4.15 ± 0.30	5.73 (4.17,8.39)	**<0.001**
**IgG Index**	0.69 (0.60,0.84)	0.68 (0.54,0.85)	0.779	0.70 (0.63,0.85)	0.73 ± 0.05	0.664
**24h intrathecal IgG synthesis rate, (mg/24h)**	1.17 (0,5.78)	1.18 (0,5.77)	0.925	0.10 (0.00,3.30)	1.81 (0.00,7.45)	0.183
**Type I isoelectric focusing pattern, n (%)**	51/71 (70.8)	36/54 (66.7)	0.617	25/38 (65.8)	26/34 (76.5)	0.32
**Type II isoelectric focusing pattern, n (%)**	15/72 (20.8)	12/54 (22.2)	0.851	12/38 (31.6)	3/34 (8.8)	**0.018**
**Type IV isoelectric focusing pattern, n (%)**	6/72 (8.3)	6/54 (11.1)	0.599	1/38 (2.6)	5/34 (14.7)	0.155
Imaging and electrophysiology						
**Cortex, n (%)**	9/87 (10.3)	7/66 (59)	0.958	4/46 (8.7)	5/41 (12.2)	0.855
**Frontal lobe, n (%)**	57/87 (65.5)	43/66 (65.2)	1	32/46 (69.6)	25/41 (61.0)	0.4
**Parietal lobe, n (%)**	49/87 (56.3)	39/66 (59.1)	0.731	30/46 (65.2)	19/41 (46.3)	0.076
**Temporal lobe, n (%)**	31/87 (35.6)	25/66 (37.9)	0.775	23/46 (50.0)	8/41 (19.5)	**0.003**
**Occipital lobe, n (%)**	21/87 (24.1)	19/66 (28.8)	0.517	17/46 (37.0)	4/41 (9.8)	**0.003**
**Periventricular, n (%)**	40/87 (46.0)	30/66 (45.5)	0.949	22/46 (47.6)	18/41 (43.9)	0.714
**Basal ganglia, n (%)**	18/87 (20.7)	15/66 (22.7)	0.762	15/46 (32.6)	3/41 (7.3)	**0.004**
**Diencephalon, n (%)**	19/87 (21.8)	16/66 (24.2)	0.726	13/46 (28.3)	6/41 (14.6)	0.125
**Brainstem, n (%)**	30/87 (34.5)	23/66 (34.8)	0.962	18/46 (39.1)	12/41 (29.3)	0.334
**Cerebellum, n (%)**	12/87 (13.8)	10/66 (15.2)	0.813	8/46 (17.4)	4/41 (9.8)	0.303
**Long segment, n (%)**	33/68 (48.5)	25/50 (50.0)	0.875	17/35 (48.6)	16/32 (50.0)	0.907
**Cervical cord, n (%)**	38/68 (55.9)	28/50 (56.0)	0.99	21/35 (60.0)	16/32 (50.0)	0.411
**Thoracic cord, n (%)**	36/68 (52.9)	28/50 (56.0)	0.742	20/35 (57.1)	16/32 (50.0)	0.558
**Lumbosacral spinal cord, n (%)**	2/68 (2.9)	2/50 (4.0)	1	2/35 (5.7)	0/32 (0)	0.493
**Circular cone/cauda equina, n (%)**	1/68 (1.5)	0/50 (0)	1	0/35 (0)	1/32 (3.1)	0.478
**VEP abnormal, n (%)**	29/48 (60.4)	23/35 (65.7)	0.622	21/27 (77.8)	8/21 (38.1)	**0.005**
**ERG abnormal, n (%)**	13/24 (54.2)	10/16 (62.5)	0.601	11/20 (55)	2/4 (50)	1
**Papilledema, n (%)**	19/43 (44.2)	14/31 (45.2)	0.934	11/29 (37.9)	8/14 (57.1)	0.235
**OCT abnormal, n (%)**	10/23 (43.5)	7/15 (46.7)	0.847	5/12 (41.7)	5/11 (45.5)	1
Treatment						
**Steroid only, n (%)**	44/91 (48.4)	39/69 (56.5)	0.306	23/47 (48.9)	21/44 (47.7)	0.908
**Steroid +IVIG, n (%)**	22/91 (24.2)	19/69 (27.5)	0.63	17/47 (36.2)	5/44 (11.4)	**0.006**
**Add immunosuppressive, n (%)**	21/91 (23.1)	8/69 (11.6)	0.062	6/47 (12.8)	15/44 (34.1)	0.16
**Steroid more than 5 weeks, n (%)**	76/91 (83.5)	59/69 (85.5)	0.731	41/47 (87.2)	35/44 (79.5)	0.323
Course and Prognosis						
**Length of stay (day)**	16 (12,21)	17 (13,23)	0.425	16 (10,20)	16 (11.5,20.5)	0.572
**Course (month)**	37.0 (27.0,50.0)	32.6 ± 1.6	**0.05**	49.0 (36.0,66.8)	50.8 (37.5,108.5)	0.745
**EDSS at admission (score)**	4 (3,5)	4 (3,5)	0.428	4 (3,5)	3.83 ± 0.32	0.553
**EDSS at discharge (score)**	1 (0,2.5)	1 (0,2.25)	0.797	1 (0,2)	2 (0,4.75)	**0.007**
**EDSS at follow-up (score)**	0 (0,1)	0 (0,1)	0.86	0 (0,1)	0 (0,1)	0.56
**mRs at admission (score)**	2 (1,4)	2 (1,4)	0.766	1 (1,3)	2 (1,4)	0.335
**mRS at discharge (score)**	1 (0,1)	1 (0,1)	0.84	1 (0,1)	1 (0,2)	**0.006**
**mRS at follow-up (score)**	1 (0,2)	1 (0,2)	0.802	0 (0,1)	0.5 (0,1)	0.243
**Relapse, n (%)**	44/91 (48.4)	22/69 (31.9)	**0.036**	26/47 (55.3)	18/44 (40.9)	0.169
Onset phenotype						
**Isolated ON, n (%)**	13/91 (14.3)	11/69 (15.9)	0.771	7/47 (14.9)	6/44 (13.6)	0.864
**Isolated TM, n (%)**	14/91 (15.4)	11/69 (15.9)	0.923	1/47 (2.1)	13/44 (29.5)	**<0.001**
**Isolated ADEM, n (%)**	28/91 (30.8)	23/69 (33.3)	0.73	20/47 (42.6)	8/44 (18.2)	**0.012**
**Isolated CE, n (%)**	5/91 (5.5)	4/69 (5.8)	1	3/47 (6.4)	2/44 (4.5)	1
**Isolated BS, n (%)**	4/91 (4.4)	3/69 (4.3)	1	3/47 (6.4)	1/44 (2.3)	0.657
**Mixed, n (%)**	27/91 (29.7)	17/69 (24.6)	0.48	13/47 (27.7)	14/44 (31.8)	0.664

The parts in bold indicate statistically significant differences (p<0.05).

In the whole cohort, 44 patients were tested for serum viral antibodies: cytomegalovirus had 35 positive cases (2 IgM^+^/IgG^+^, 33 IgM^-^/IgG^+^); Epstein-Barr virus had 37 positive cases (2 IgM^+^/IgG^+^, 35 IgM^-^/IgG^+^); human parvovirus B-19 had 3 positive cases (1 IgM^+^/IgG^+^, 2 IgM^-^/IgG^+^); adenovirus had 2 positive cases (1 IgM^+^/IgG^-^, 1 IgM^-^/IgG^+^), and 11 cases of coxsackievirus, 7 cases of measles virus, and 3 cases of herpes simplex virus were found to be IgM^-^/IgG^+^. Sixty-one patients had CSF tested for viruses, of whom two tested positive for herpes zoster virus IgG.

**Figure 3:**
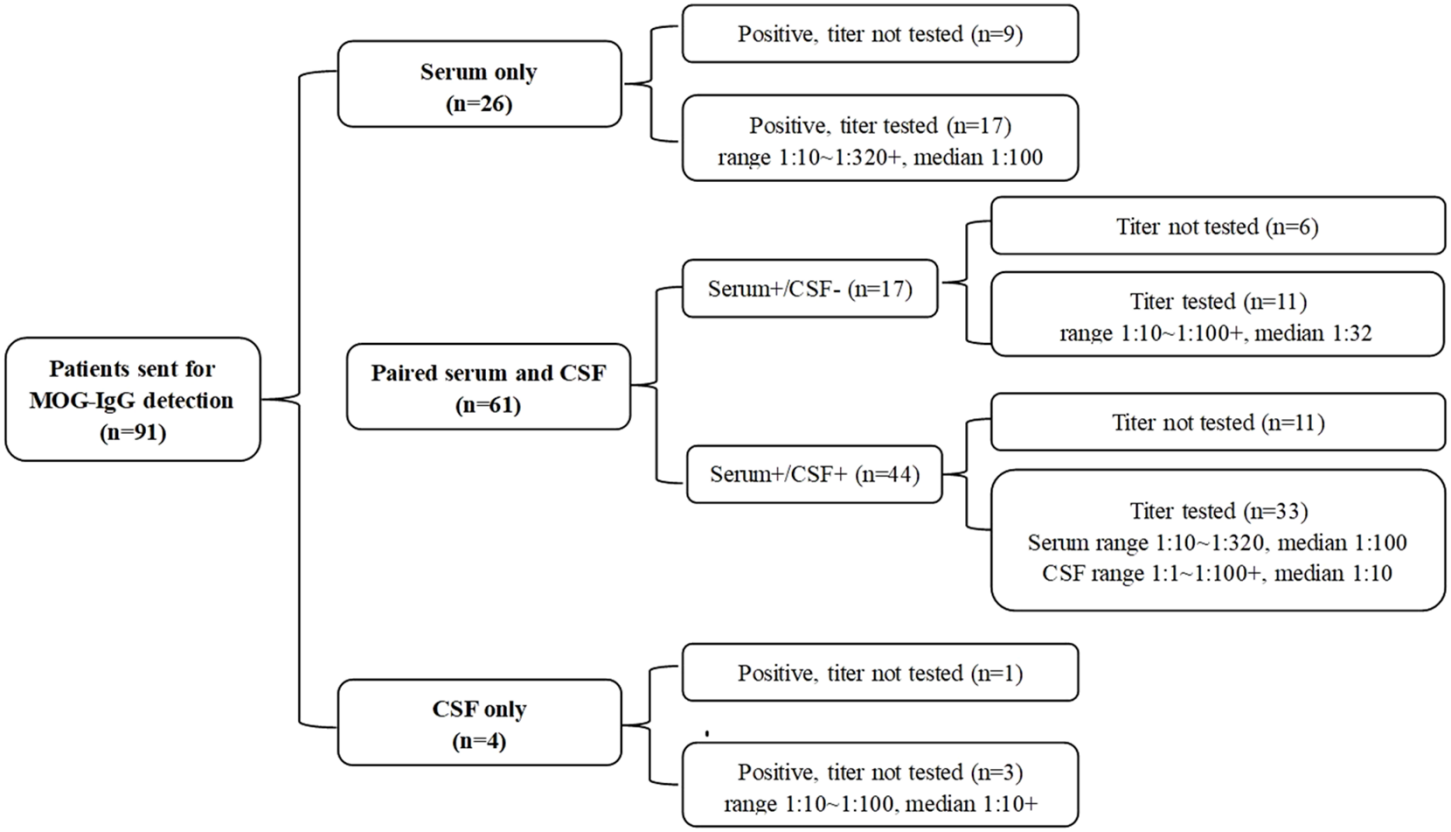
MOG-IgG test results of 91 patients.

Of the 91 patients, 26 patients only underwent serum MOG antibody detection, 4 patients only underwent CSF MOG antibody detection, and the remaining 61 patients’ serum and cerebrospinal fluid samples were tested at the same time. The results and titers are shown in **Figure 3**.

A total of 87 patients in the whole cohort underwent head MRI. The most frequently affected regions were the frontal lobe (66%, 57/87), parietal lobe (56%, 49/87), and paraventricular lobe (46%, 40/87). Spinal cord MRI was performed in 68 patients, of whom 49% (33/68) had multi-segment involvement. The most common site of involvement was the cervical spinal cord (56%, 38/68), followed by the thoracic spinal cord (53%, 36/68), and lumbar/sacral cord (3%, 2/68). Only one patient had conus and cauda equina abnormalities.

Among the 28 patients who underwent EEG examination, 50% (14/28) had abnormal findings, which was found in 83.3% (5/6) of Mixed (all ADEM combined with ON), 50% (1/2) of ON, 43.8% (7/16) of ADEM, 33.3% (1/3) of CE and 0% (0/1) of TM. Among them, only 1 case of CE showed epileptic waves: 2–3 Hz sharp slow waves were observed in the left anterior temporal-middle temporal region during sleep. Other EEG abnormalities were not specific. Details of the patient’s clinical symptoms, phenotype, MRI and EEG are shown in **Supplementary Table 1**.

### 3.3 Comparison of children and adults

The median age at onset was 8 years (range: 1–17 years) in children and 34 years (range: 18–79 years) in adults. The peak ages of presentation were between 5 and 10 years of in children, and 30 and 35 years in adults (**Figure 2A**).

The clinical characteristics, laboratory test results, imaging, and electrophysiological findings in children and adults are compared in **Figure 2C** and **Table 1**. The proportion of children with ADEM was significantly higher than that of adults (43% versus 18%, p = 0.012), whereas the proportion of adults with TM was significantly higher than in adults (29.5% versus 2.1%, p < 0.001). The incidence of fever, nausea and vomiting, seizures, and disorders of consciousness was higher in children than in adults. Conversely, the incidence of paresthesia was higher in adults than in children.

Compared with adults, children had a significantly higher rate of ICU admission (32% versus 7%, p = 0.005) and were significantly more likely to receive steroid and IVIG treatment during hospitalization (36% versus 11.4%, p = 0.006). Children had better short-term outcomes at discharge than adults (median [IQR] EDSS: 1 [0–1] versus 2 [0–4.75], p = 0.007; median [IQR] mRS: 1 [0–1] versus 1 [0–2], p = 0.006). The long-term prognosis and risk of recurrence did not differ significantly between adults and children.

### 3.4 Comparison of the four clinical phenotypes

The CSF characteristics, treatment regimens, severity, and short-term prognosis of patients with different clinical phenotypes (isolated ON, isolated TM, brain, and mixed) are compared in **Table 2**. Patients with TM were significantly older than patients with other phenotypes (p < 0.001). The EDSS (p = 0.031) score on admission was higher in patients with TM, whereas the EDSS (p = 0.002) and mRS (p < 0.001) scores on discharge were lower in patients with the brain phenotype. During hospitalization, the immunotherapy regimen did not differ significantly among the four phenotypes.

**Table 2:** Demographic and clinical characteristics of patients according to the clinical phenotype.

Variables	Isolated ON (n=13)	Isolated TM (n=14)	Brain (n=37)	Mixed (n=27)	P
**Female, n (%)**	5/13 (38.5)	4/14 (28.6)	15/37 (40.5)	15/27 (55.6)	0.372
**Age of onset (years)**	20.1 ± 3.9	42.9 ± 4.9	10.0 (5.5,23.5)	18.0 (10.0,33.0)	**<0.001**
**CSF cell count (×10^6/L)**	5.0 (3.5,51.8)	14.0 (2.0,14.0)	37.0 (16.0,76.0)	26.0 (4.5,52.0)	0.199
**CSF glucose (mmol/L)**	4.21 ± 0.31	2.98 ± 0.12	3.19 (2.76,3.52)	3.34 (2.91,3.57)	**0.010**
**CSF protein (mg/L)**	241.0 (161.8,385.1)	399.2 (359.5,753.6)	327.0 (271.0,429.6)	293.2 (250.1,388.0)	**0.009**
**CSF immunoglobulin IgG (mg/L)**	16.80 (9.53,24.85)	67.86 ± 11.785	31.15 (24.63,50.65)	29.17 ± 2.23	**<0.001**
**CSF albumin (mg/L)**	117.6 (94.8,181.1)	301.7 (217.1,387.0)	188.4 (145.0,260.1)	191.2 ± 11.4	**<0.001**
**QIgG**	1.72 ± 0.25	6.37 ± 0.74	3.36 (2.27,4.71)	2.86 ± 0.245	**<0.001**
**QAlb**	2.96 (2.10,3.88)	8.32 ± 0.95	4.51 (3.51,5.82)	4.39 ± 0.27	**<0.001**
**IgG Index**	0.53 ± 0.07	0.78 ± 0.05	0.80 ± 0.05	0.64 (0.60,0.71)	**0.008**
**24h intrathecal IgG synthesis rate (mg/24h)**	0.00 (-4.46,0.14)	10.16 ± 2.33	2.51 (0.00,5.88)	0.55 (0.00,1.86)	**<0.001**
**Type I isoelectric focusing pattern, n (%)**	7/9 (77.8)	9/11 (81.8)	18/28 (64.3)	17/24 (70.8)	0.698
**Type II isoelectric focusing pattern, n (%)**	1/9 (11.1)	1/11 (9.1)	9/28 (32.1)	4/24 (16.7)	0.277
**Type IV isoelectric focusing pattern, n (%)**	1/9 (11.1)	1/11 (9.1)	1/28 (3.7)	3/24 (12.5)	0.688
**Damage of BBB, n (%)**	1/9 (11.1)	8/11 (72.7)	12/28 (42.9)	6/24 (25.0)	**0.015**
**Steroid only, n (%)**	8/13 (61.5)	6/14 (42.9)	15/37 (40.5)	15/27 (55.6)	0.468
**Steroid +IVIG, n (%)**	2/13 (15.4)	5/14 (35.7)	11/37 (29.7)	4/27 (14.8)	0.324
**Add immunosuppressive, n (%)**	3/13 (23.1)	3/14 (21.4)	7/37 (18.9)	8/27 (29.6)	0.793
**Steroid more than 5 weeks, n (%)**	12/13 (92.3)	12/14 (85.7)	27/37 (73.0)	25/27 (92.6)	0.146
**EDSS at admission (score)**	3.19 ± 0.22	5.18 ± 0.56	4.00 (2.75,5.00)	4.04 ± 0.39	**0.031**
**EDSS at discharge (score)**	2 (2,2)	5.25 (1.00,6.00)	0.00 (0.00,1.00)	1.00 (0.00,2.50)	**<0.001**
**mRs at admission (score)**	2 (1,2)	4 (1,4)	1 (1,2)	1 (1,4)	0.092
**mRS at discharge (score)**	1 (1,1.5)	1.93 ± 0.34	0 (0,1)	1 (0,1)	**<0.001**

The parts in bold indicate statistically significant differences (p<0.05).

**Figure 4:**
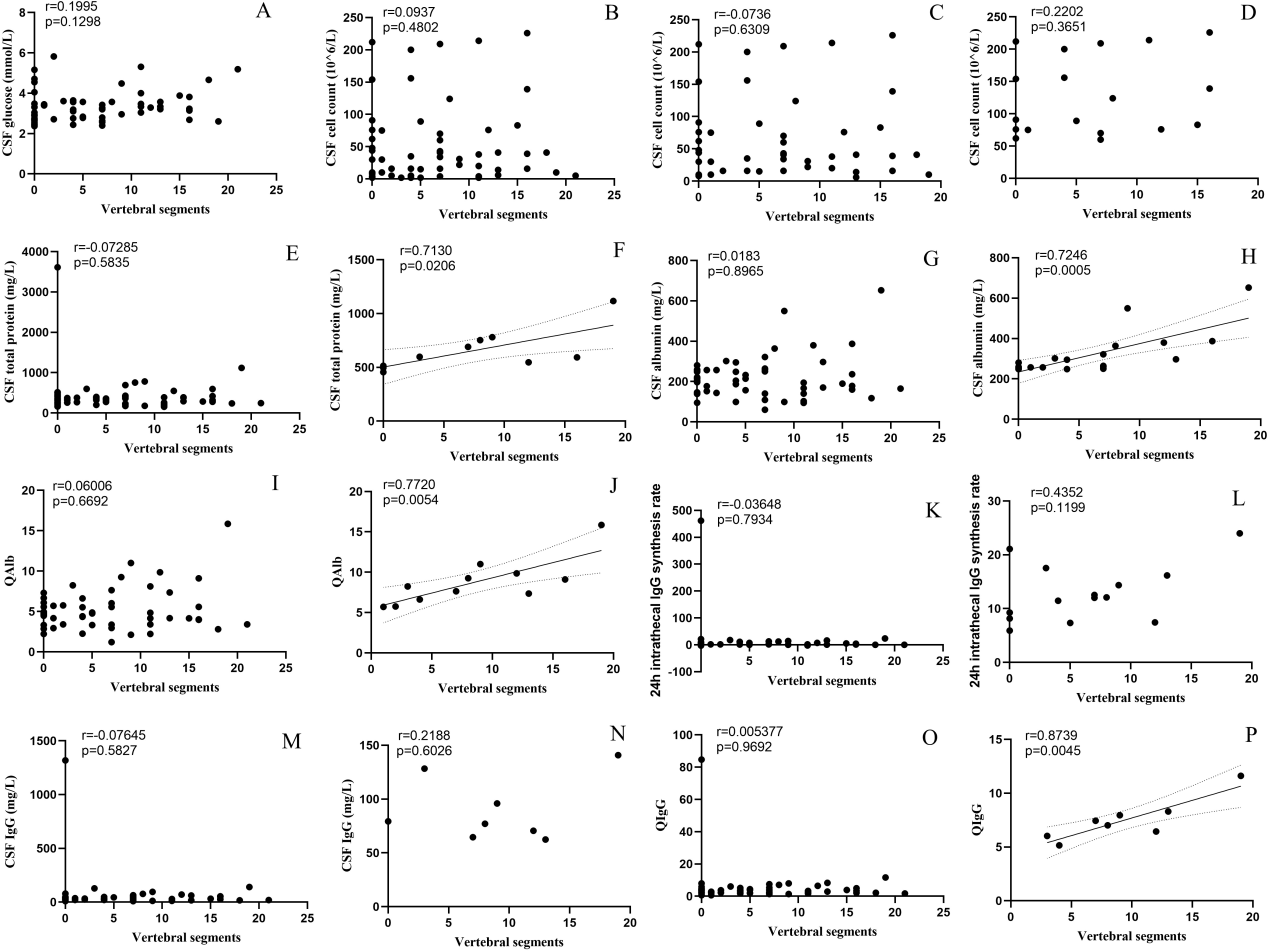
Correlation between CSF parameters and spinal cord lesion segments. (**(A)** Glucose, **(B)** Cell count, **(C)** Cell count>5×10^6^/L, **(D)** Cell count>50×10^6^/L, **(E)** Total protein, **(F)** Total protein>450mg/L, **(G)** Albumin, **(H)** Albumin>450mg/L, **(I)** QAlb, **(J)** increased Qalb*, **(K)** 24 h intrathecal IgG synthesis rate, **(L)** 24 h intrathecal IgG synthesis rate>3.3 mg/24 h, **(M)** IgG, **(N)** IgG>40mg/L, **(O)** QIgG, **(P)** QIgG>0.7). *:≤ 15 years old: >5.0×10^-3^; 16~40 years old: >6.5×10^-3^; 40~60 years old: >8×10^-3^; >60 years old: >9×10^-3^. CSF, cerebrospinal fluid; IgG, immunoglobulin G; QAlb, albumin quotient; QIgG, immunoglobulin G quotient.

The incidence of BBB damage and the CSF test results in the TM group differed significantly from those of the other three groups. To further explore the correlation between CSF test results and spinal cord injury, we performed a correlation analysis between the number of spinal segments involved, and the CSF test results (**Figure 4**). After removing significant outliers, the CSF total protein level (r=0.713, p=0.021), Alb level (r = 0.725, p < 0.001), QAlb (r = 0.772, p = 0.005), and QIgG (r = 0.874, p = 0.005) that exceed the normal range were all positively correlated with the number of spinal segments involved.

### 3.5 Treatment

In the whole cohort, 69% (62/90) patients were treated with intravenous steroids alone, 27% (24/90) were treated with steroids and IVIG, 3 were treated with IVIG alone, and 1 was treated with steroids, IVIG and plasma exchange during hospitalization. After discharge, 87 patients who were treated with intravenous steroids in the acute phase were treated with maintenance oral steroids, of whom 87.4% (76/87) were treated with steroids for more than 5 weeks. In the whole cohort, 23.1% (21/91) patients were treated with immunosuppressants, including mycophenolate mofetil (14 patients), azathioprine (3 patients), tacrolimus (2 patients), methotrexate (1 patient), and ofatumumab (1 patient). In the incident cohort, 67.6% (46/68) patients were treated with steroids alone, 27.9 (19/68) were treated with steroids and IVIG, 2 were treated with IVIG alone, and 1 was treated with steroids, IVIG, and plasma exchange. After discharge, 8 patients received additional immunosuppressants, including mycophenolate mofetil (6 patients), azathioprine (1 patient), and methotrexate (1 patient).

### 3.6 Risk factors for recurrence

**Figure 5:**
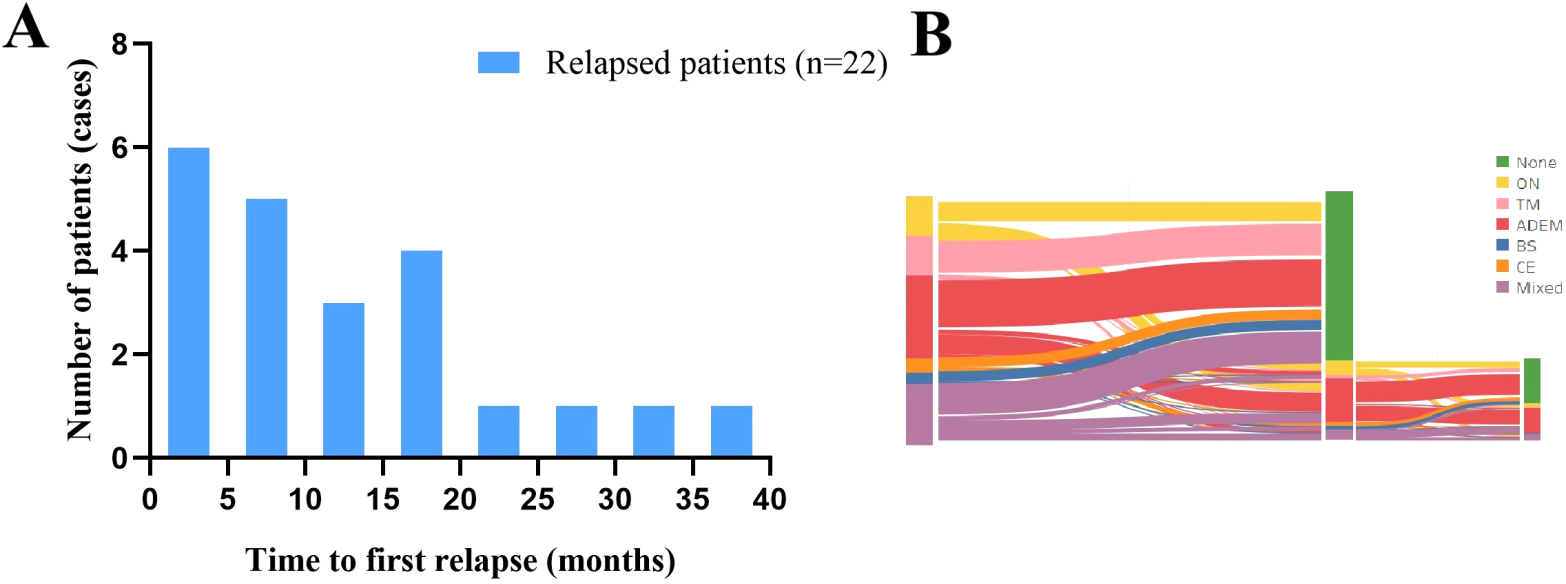
Characteristics of recurrence in the incident cohort. **(A)** Time to the first recurrence in the incident cohort. **(B)** Distribution of clinical phenotypes and changes in the incident cohort. ADEM, acute disseminated encephalomyelitis; BS, brainstem syndrome; CE, cortical encephalitis; MOGAD, myelin oligodendrocyte glycoprotein antibody-associated disease; ON, optic neuritis, TM, transverse myelitis.

To avoid overestimating the recurrence rate, we only analyzed the data of the 69 patients in the incident cohort. They were followed up for a median of 34 months, and 31.9% of the patients experienced recurrence after 9 months (range, 1–36 months) (**Figure 5A**), and the changes in clinical phenotypes in the incident cohort is shown in **Figure 5B**. The variables evaluated included sex, age at onset, clinical presentation, routine cerebrospinal fluid, isoelectric focusing pattern, and treatment regimen. Headache (OR: 3.08, 95% CI: 1.08–8.79, p = 0.035) was associated with risk of recurrence, whereas steroid maintenance therapy for longer than 5 weeks (OR: 0.25, 95% CI: 0.06–0.995, p = 0.049) was associated with a reduced the risk of recurrence (**Supplementary Table 2**). In the multivariable regression analysis, visual impairment was an independent risk factor for recurrence (OR: 4.22, 95% CI: 1.24–14.38, p = 0.022) (**Table 3**).

**Table 3:** Logistic regression analysis of factors associated with recurrence.

Variable	Univariate Analysis		Multivariate Analysis	
	OR (95% CI)	P	OR (95% CI)	P
**Visual impairment**	2.868 (0.991-8.301)	0.052	4.215 (1.236-14.377)	**0.022**
**Headache**	3.081 (1.08-8.789)	**0.035**	3.209 (0.989-10.419)	0.052
**Steroid more than 5 weeks**	0.248 (0.062-0.995)	**0.049**	0.211 (0.043-1.026)	0.054

The parts in bold indicate statistically significant differences (p<0.05).

### 3.7 Risk factors for poor long-term prognosis

To evaluate the impact of clinical characteristics and treatment regimens on patient prognosis, we included clinical manifestations, cerebrospinal fluid, EDSS score at discharge, total number of episodes, and treatment in the analysis. Single-variable and multivariable regression analyses were performed on the 69 patients in the incidence cohort. Logistic regression analysis showed that higher EDSS score at discharge (OR: 5.05, 95% CI: 1.27–20.07, p = 0.021), and more episodes (OR: 9.24, 95% CI: 1.35–63.11, p = 0.023) were associated with poor prognosis, and type I isoelectric focusing pattern (no oligoclonal bands [OB] in serum and cerebrospinal fluid) (OR: 0.004, 95% CI: 0.000–0.40, p = 0.019) and steroid use for > 5 weeks (OR: 0.001, 95% CI: 0.000–0.34, p = 0.019) were associated with favorable prognosis (**Table 4**). In the unadjusted logistic regression analysis, vomiting was associated with poor prognosis (OR: 5.21, 95% CI: 1.28–21.16, p = 0.021) was; however, no significant association was observed between vomiting and prognosis in the multivariable regression analysis (**Supplementary Table 3**).

**Table 4:** Logistic regression analysis of factors associated with poor long-term prognosis.

Variable	Univariate Analysis		Multivariate Analysis	
	OR (95% CI)	P	OR (95% CI)	P
**Nausea/vomiting**	5.208 (1.282-21.163)	**0.021**	24.684 (0.573-1063.04)	0.095
**Type I isoelectric focusing pattern**	0.196 (0.048-0.802)	**0.023**	0.004 (0.000-0.402)	**0.019**
**EDSS at discharge**	1.529 (1.138-2.055)	**0.005**	5.050 (1.270-20.074)	**0.021**
**Total number of episodes**	2.007 (1.188-3.393)	**0.009**	9.235 (1.352-63.105)	**0.023**
**Steroid more than 5 weeks**	0.090 (0.02-0.399)	**0.002**	0.001 (0.000-0.339)	**0.019**

The parts in bold indicate statistically significant differences (p<0.05).

## 4 Discussion

This study describes the clinical characteristics of patients with MOGAD in a single center in central China. In this study, we compared the whole cohort and the incident cohort, children and adults, and different clinical phenotypes. In addition, we evaluated the risk factors that may affect recurrence and long-term prognosis to aid in the diagnosis and treatment of MOGAD.

The male-to-female ratio, clinical phenotypes, and prodromal symptoms observed in our study were similar to those reported previous studies (23–27). In contrast to previous studies (12, 28), the ADEM phenotype was the most common phenotype, followed by the mixed, TM, and ON groups. This difference may be due to the inclusion of patients with only optic nerve damage in the ON group, whereas patients with optic nerve involvement accompanied by brain or spinal cord involvement were included in the mixed group, and all 27 patients with Mixed phenotype had symptoms of optic nerve damage, including 20 patients with ON and ADEM, 4 patients with ON and BS, and 3 patients with ON and TM, so 44% of the patients had symptoms of optic nerve damage. This inference is supported by the proportion of patients with visual impairment in this study. In addition, in our study, the ratio of children to adults was close to 1:1, which was significantly higher than that in previous studies (28, 29), and ADEM is more common in children (30), which may also explain the relatively high prevalence of the ADEM phenotype.

In the comparison of child and adult cohorts, ADEM was more common in children, whereas TM was more common in adults, which is consistent with previous studies (29). Although the proportion of patients with VEP abnormalities was higher in children, there was no significant difference in the proportions of visual impairment, ERG abnormalities, optic disk edema, and OCT abnormalities between children and adults. The difference in VEP abnormalities between children and adults may be attributable to the small sample size. As VEP is useful in infants and children who are too young to talk, a higher proportion of children undergo VEP investigation; therefore, VEP abnormalities are more likely to be detected in children than in adults (31). Previous studies have shown that positive CSF-restricted MOG antibodies are associated with MOGAD in adults (32), while Olive et al. suggested that the significance of positive CSF-restricted MOG antibodies in children is different from that in adults, suggesting that they are associated with MS, but they did not analyze the isoelectric focusing patterns (33). In this study, type II isoelectric focusing pattern was more likely to be detected in children, suggesting that intrathecal MOG antibody synthesis may be more active in children, but all patients with type II isoelectric focusing pattern had higher serum antibody titers than CSF titers, and no restricted CSF MOG antibody was found in our patients. It is further speculated that intrathecal synthesis in MOGAD in children is accompanied by systemic immune activation, but not by restricted CNS intrathecal synthesis. Greco study, which corrected the effect of BBB damage and used “AIMOG” to evaluate the intrathecal synthesis of “ITS”, concluded that patients with intrathecal synthesis were more likely to have aggressive disease phenotypes, were more likely to use IVIg/PLEX in the acute phase, and were associated with poor prognosis (34). In our study, there was no significant difference in IgG index, 24-hour IgG synthesis rate and BBB damage rate between children and adults, and the invasive phenotype was more common in adults. Although the sample size limitation cannot be ruled out, this study may also suggest that there is an age-related transient intrathecal immune response in MOGAD, which does not accumulate to significant increase in IgG index or chronic damage. In addition, there may be different immunoglobulin subtypes in children than in adults, which are more easily detected by isoelectric focusing (35), and their pathological effects are weaker than those in adults. It is speculated that future studies combined with longitudinal isoelectric focusing pattern analysis, IgG subtype detection and MOG epitope localization are needed to further reveal the essential differences in MOGAD immune responses between children and adults. The proportion of children treated with steroids and immunoglobulins during hospitalization was significantly higher than that of adults, and the EDSS score and mRS on discharge were more favorable in children than in adults. The combination of steroid and IVIG therapy may improve the short-term prognosis of patients (36). In addition, because children have a better nerve repair function than adults (37), and adults are more likely to present with TM, this may also lead to a better short-term prognosis in children than in adults.

Patients in the isolated TM group had a higher incidence of BBB damage than those in other phenotype groups, and BBB damage was associated with spinal cord lesions, which is consistent with previous findings (38). To examine BBB impairment associations in MOGAD, we analyzed CSF biomarkers against spinal lesion extent. Spinal cord involvement severity positively correlated with BBB disruption degree.

After a median follow-up of 34 months, 32% of the 69 of the patients with a first episode had a recurrence, with a median time to recurrence of 9 months, which is similar to that reported in previous studies (29). In our study, headache and visual impairment were associated with an increased risk of recurrence. Although we did not find a higher risk of recurrence in the ON group, possibly because of group differences, many previous studies have pointed out that ON is the most common clinical manifestation at the time of recurrence (18, 24), this is consistent with our finding that vision loss increases the risk of recurrence. In our study, headache was a risk factor for recurrence. First, headache may indicate the production of more pain-promoting brain-derived neurotrophic factor (BDNF), cytokines and chemokines (IL-1β, IL-17 and TNF) (39–41), that is, a more severe inflammatory response, which may not only lead to incomplete myelin repair, but also lead to more severe inflammatory response. It can also form a chronic subclinical inflammatory microenvironment in the central nervous system, which in turn reduces the threshold of immune homeostasis and increases the risk of MOGAD recurrence (42). In addition, headache may indicate more pronounced meningeal inflammation (43–46), and the meninges, which act as a barrier for immune cells to enter the CNS (47, 48), may be involved by promoting the formation of lymphoid follicular structures (49), providing a local microenvironment for B cell survival (50), and by abnormal drainage of meningeal lymphatic vessels. It causes difficulty in antibody clearance (51) and affects brain myelination (52), which in turn increases the risk of relapse. In addition, some studies have pointed out that MOG-related ON can have intra-orbital and peri-optic striate inflammation, which may involve the meninges and communicating fibers around the optic nerve and lead to headache (53). Furthermore, 50% of the 38 patients who presented with headache also had optic neuritis-like features; therefore, we cannot exclude the possibility that the prediction of recurrence by headache may be influenced by optic nerve damage. Due to the limited sample size, further studies are required to confirm our findings. In this study, a longer duration of steroid use was associated with a lower risk factor for recurrence, which is consistent with the findings of previous studies (54, 55).

In the prognostic analysis, short-term improvement of neurological function and the cumulative number of episodes affected the long-term outcomes of patients, suggesting that timely and effective immunotherapy may contribute to improved prognosis, and poor outcomes are associated with the number of recurrences, which further emphasizes the clinical value of preventing disease recurrence. For patients with a first episode, the absence of increased intrathecal synthesis levels may indicate a better long-term prognosis (34, 56, 57). Regarding the effect of treatment strategy, the appropriate duration of steroid maintenance has been shown to have a dual benefit: previous studies have shown that steroid therapy for >5 weeks at a first exacerbation reduces the risk for recurrence (55), but this study reinforces that longer duration of steroid therapy also significantly improves long-term outcomes. Previous studies have shown that patients with MOGAD have a rapid and dramatic response to steroids, and longer treatment courses may be associated with reduced recurrence (58). Notably, reduced risk of recurrence within the first year after onset is also associated with reduced risk of long-term recurrence (59), further emphasizing the importance of aggressive intervention in the early stages of MOGAD disease. The recommended duration of steroid therapy varies, with recommendations ranging from 5 weeks and 6 months (17, 55, 60). Trewin et al. first recommended prednisone at a dose of 12.5 mg per day at the time of onset (0.16 mg per kilogram per day in children) for at least 3 months to delay the first relapse and to avoid adverse events associated with steroid exposure during the initial phase of MOGAD (61). Although the present study found an improvement in the long-term prognosis of patients with newly diagnosed MOGAD with longer duration of steroid use, there were shortcomings compared with Trewin’s study. This study did not provide in-depth exploration of the precise duration of steroid maintenance and only preliminary prognostic observations. It did not provide in-depth analysis of the quantitative relationship between steroid use and improved outcomes. In the future, it is necessary to further study the association between steroid dosage and prognosis, and construct a model to accurately predict the prognosis effect. At the same time, patients should be followed up for a long time to comprehensively evaluate the adverse reactions of long-term use of steroids, so as to provide scientific basis for clinical rational drug use, improve the therapeutic effect and reduce the risk.

Our study has some limitations. First, it is a retrospective study, and some clinical data were missing. Moreover, most patients without a recurrence, did not have follow-up data on antibody levels; therefore, the changes in antibody levels were not analyzed. Second, our study population was from a single center, which limited the source and number of patients and may limit the generalizability of the findings. Large, multicenter, prospective cohort studies are needed to confirm our findings.

## Data Availability

The raw data supporting the conclusions of this article will be made available by the authors, without undue reservation.
