# Supplementary material for: Clinical characteristics and factors associated with recurrence and long-term prognosis in patients with MOGAD
Source: Front Immunol. 2025 May 8;16:1535571. doi: 10.3389/fimmu.2025.1535571 (PMC12095161; doi:10.3389/fimmu.2025.1535571)
Supplement: Supplementary file 1 [file Table1.docx]

Supplementary Material

# Supplementary Data

Supplementary Material should be uploaded separately on submission. Please include any supplementary data, figures and/or tables.

Supplementary material is not typeset so please ensure that all information is clearly presented, the appropriate caption is included in the file and not in the manuscript, and that the style conforms to the rest of the article.

# Supplementary Tables

**Supplementary Table 1** Details of Patients with Abnormal EEG

| Patient no. sex/age of onset | Summary clinical symptoms | Clinical syndroms | MRI findings | Electroencephalogram |
| --- | --- | --- | --- | --- |
| 01. M/55 | Headache, dizziness, nausea, seizure | ADEM | **Brain**: T2-hyperintense lesions in the bilateral frontal and parietal lobes, bilateral periventricular regions, left basal ganglia, and right hippocampal region (multiple patchy and punctate) | Interictal period: Mildly increased slow-wave activity in the bifrontal regions |
| 02. M/15 | Fever, headache, dizziness, nausea, vomiting, bowel/bladder disturbance, paresthesia, limb weakness, seizure, dysmnesia, consciousness disorders, mental disorders, dyskinesia, speech disorders, visual impairment | Mixed (ADEM and ON) | **Brain**: T2-hyperintense lesions in the bilateral frontal base, bilateral temporal hippocampus, bilateral basal ganglia, bilateral thalamus and optic chiasm (patchy), left lateral ventricle paraventricular (small patchy). | The whole conduction showed intermittent 1-2Hz medium-amplitude δ activity, which was basically symmetrical bilaterally and prominent in the central area. The awake and sleep cycles could not be distinguished. |
| 03. M/27 | Fever, headache, bowel/bladder disturbance, seizure, consciousness disorders, mental disorders，dyskinesia | ADEM | **Brain**: T2-hyperintense lesions in the right frontal lobe, right periventricular, and left frontal lobe (patchy pattern), with cortical swelling in the right cerebral hemisphere | Slow waves: Paroxysmal activities of abundant medium to high amplitude 2-3Hz δ waves were observed in the bifrontal regions. |
| 04. M/3 | Fever, headache, somnipathy, bowel/bladder disturbance, limb weakness, seizure, consciousness disorders, dyskinesia，speech disorders，choking on liquids，dysphagia | ADEM | **Brain**: T2-hyperintense lesions in the left frontoparietal occipital local cortex and subcortical and right basal ganglia (patchy) | During sleep, slightly increased slow wave activity predominantly in the bilateral parietal regions, right occipital region, and right anterior temporal region was observed, with a frequency of approximately 2.5Hz, spreading to adjacent leads. |
| 05. M/13 | Headache, seizure, consciousness disorders, visual impairment | Mixed (ADEM and ON) | **Brain**: normal.  **Optic nerve**: normal. | In the right occipital lobe, there were many medium-high amplitude medium-long range intermittent discharges of δ and θ waves for 2-5 hours. |
| 06. F/11 | Visual impairment, ocular movement pain, visual field defects | ON | **Brain**: normal.  **Optic nerve**: T2 SPIR-hyperintense lesions in bilateral optic nerves and the right optic nerve was thicken. | The background rhythm was slowed. |
| 07. M/20 | Seizure, consciousness disorders | CE | **Brain**: T2-hyperintense lesions in the right frontal cortex. | During sleep, sharp and slow wave complexes (2.0-3.0Hz) were observed in the left anterior to mid-temporal regions. During Clinical Seizure Onset (Ictal Phase): Progressive acceleration in frequency and gradual increased in amplitude of epileptiform discharges. |
| 08. F/15 | Dysmnesia, mental disorders | ADEM | **Brain**: T2-hyperintense lesions in the bilateral frontal lobes and bilateral periventricular (multiple patchy). T1WI FS +C-enhanced lesions in the bilateral frontal lobes and bilateral lateral ventricles (multiple patchy). | Moderate diffused abnormalities. |
| 09. M/8 | Fever, nausea, vomiting, seizure, consciousness disorders | ADEM | **Brain**: T2-hyperintense lesions in the bilateral lateral ventricle posterior horn, bilateral parieto-occipital lobe local cortex and right hippocampus. | During sleep, intermittent slow waves of medium amplitude were observed in the right frontal area, central area and forehead. |
| 10. M/18 | Fever, headache, bowel/bladder disturbance, seizure, consciousness disorders, speech disorders，visual impairment | Mixed (ADEM and ON) | **Brain**: T2-hyperintense lesions in the left frontotemporal lobe (patchy). T1WI+C-enhanced lesions in the left frontal lobe (patchy).  **Optic nerve**: T2-the optic nerves were slightly thickened bilaterally. T1WI+C-enhanced lesions in the full length of bilateral optic nerves. | Slow waves increased in the prefrontal cortex. |
| 11. F/4 | Fever, nausea, vomiting, seizure, consciousness disorders, visual impairment | Mixed (ADEM and ON) | **Brain**: T2-hyperintense lesions in the bilateral frontoparietal, temporal and occipital lobes, bilateral lateral ventricles, bilateral basal ganglia, bilateral thalamus and medulla oblongata (multiple clusters and sheets).  **Optic nerve**: T2 FLAIR-hyperintense lesions in the bilateral retrobulbar segments of the optic nerve. | During awake state, moderate amplitude 5Hz theta activity was observed in the bilateral occipital regions during quiet, eyes-closed rest, with roughly symmetrical distribution between the left and right sides. |
| 12. M/2 | Fever, nausea, vomiting, limb weakness, seizure, consciousness disorders, dyskinesia | ADEM | **Brain**: T2-hyperintense lesions in the bilateral frontoparietal, temporal and occipital lobes, right insular lobe, bilateral basal ganglia and pons (multiple patches). | Diffuse increased in slow wave activity. |
| 13. M/3 | Fever, headache, nausea, vomiting, seizure, consciousness disorders | ADEM | **Brain**: T2-hyperintense lesions in the bilateral cerebral hemispheres, bilateral basal ganglia, thalamus and brainstem surface. **Spine**: lesions incervical cord. | Diffuse increased in slow wave activity. |
| 14. F/12 | Dizziness, somnipathy, nausea, vomiting, paresthesia, limb weakness, seizure, consciousness disorders, dyskinesia, visual field defects | Mixed (ADEM and ON) | **Brain**: T2-hyperintense lesions in the right frontal lobe (patchy). | Moderate-high amplitude slow waved in the right hemisphere. |

**Supplementary Table 2.1** Univariate Analysis of Factors Associated with a Relapsing Course

| Variables | Unidirectional course | Recurrent course | P | OR | 95%CI |
| --- | --- | --- | --- | --- | --- |
| Female | 19/47(40.4) | 9/22(40.9) | 0.97 | 0.98 | 0.350-2.747 |
| Age of onset | 20.0(8.0,40.0) | 15.5(6.75,29.25) | 0.187 | 0.98 | 0.951-1.010 |
| Clinical symptoms |  |  |  |  |  |
| Fever | 18/47(38.3) | 12/22(54.5) | 0.207 | 1.933 | 0.694-5.387 |
| Visual impairment | 17/47(36.2) | 13/21(61.9) | 0.052 | 2.868 | 0.991-8.301 |
| Ocular movement pain | 11/47(23.4) | 3/21(14.3) | 0.395 | 0.545 | 0.135-2.204 |
| Visual field defects | 6/41(14.6) | 5/19(26.3) | 0.283 | 2.083 | 0.546-7.948 |
| Headache | 15/47(31.9) | 13/22(59.1) | **0.035** | 3.081 | 1.08-8.789 |
| Dizziness | 6/47(12.8) | 5/22(22.7) | 0.298 | 2.01 | 0.540-7.483 |
| Somnipathy | 7/47(14.9) | 7/22(31.8) | 0.11 | 2.667 | 0.800-8.889 |
| Nausea/vomiting | 10/47(21.3) | 4/22(18.2) | 0.766 | 0.822 | 0.227-2.984 |
| Bowel/bladder disturbance | 13/47(27.7) | 4/22(18.2) | 0.398 | 0.581 | 0.165-2.045 |
| Paresthesia | 15/47(31.9) | 5/22(22.7) | 0.435 | 0.627 | 0.195-2.023 |
| Limb weakness | 20/47(42.6) | 9/22(40.9) | 0.897 | 0.935 | 0.334-2.613 |
| Seizure | 13/47(27.7) | 8/22(36.4) | 0.465 | 1.495 | 0.508-4.394 |
| Dysmnesia | 2/47(4.3) | 1/22(4.5) | 0.956 | 1.071 | 0.092-12.487 |
| Consciousness disorders | 14/47(29.8) | 8/22(36.4) | 0.585 | 1.347 | 0.462-3.928 |
| Mental disorders | 3/47(6.4) | 0/22(0) | 0.999 | 0 | 0 |
| Dyskinesia | 21/47(44.7) | 7/22(31.8) | 0.313 | 0.578 | 0.199-1.677 |
| CSF cell number（10^6/L） | 33.00(10.00,76.00) | 25.00(5.00,74.75) | 0.828 | 1.001 | 0.992-1.010 |
| CSF glucose (mmol/L) | 3.16(2.77,3.86) | 3.44(2.75,3.62) | 0.658 | 1.158 | 0.605-2.216 |
| CSF protein (mg/L) | 32.00(248.35,423.00) | 323.45(271.50,433.88) | 0.547 | 0.999 | 0.997-1.001 |
| CSF immunoglobulin IgG(mg/L) | 30.50(23.85,49.15) | 36.00±4.47 | 0.662 | 0.997 | 0.983-1.011 |
| CSF albumin (mg/L) | 214.00(147.20,295.95) | 178.30(145.00,252.65) | 0.358 | 0.997 | 0.992-1.003 |
| QIgG | 3.38(2.23,4.72) | 2.48(1.92,3.93) | 0.461 | 0.899 | 0.677-1.194 |
| QAlb | 4.78(3.45,7.49) | 4.15(3.40,5.65) | 0.312 | 0.879 | 0.686-1.128 |
| IgG Index | 0.71±0.05 | 0.71±0.05 | 0.936 | 0.909 | 0.088-9.344 |
| 24h intrathecal IgG synthesis rate(mg/24h) | 1.44(0.00,5.86) | 0.47(0.00,4.00) | 0.661 | 0.992 | 0.957-1.028 |
| Type Ⅰ isoelectric focusing pattern | 25/37(67.6) | 11/17(64.7) | 0.836 | 0.88 | 0.263-2.950 |
| Type Ⅱ isoelectric focusing pattern | 7/37(18.9) | 5/17(29.4) | 0.392 | 1.786 | 0.473-6.743 |
| Type Ⅳ isoelectric focusing pattern | 5/37(13.5) | 1/17(5.9) | 0.42 | 0.4 | 0.043-3.717 |
| Damage of BBB | 16/37(43.2) | 5/17(29.4) | 0.336 | 0.547 | 0.160-1.870 |
| EDSS at admission(score) | 5(3,6) | 4.46±0.33 | 0.556 | 1.083 | 0.830-1.415 |
| EDSS at discharge(score) | 1(1,5) | 1(0,4.75) | 0.964 | 0.998 | 0.897-1.109 |
| mRs at admission(score) | 0(0,1) | 0(0,1.25) | 0.566 | 1.104 | 0.787-1.548 |
| mRS at discharge(score) | 1(0,1) | 1(0,1) | 0.7 | 0.901 | 0.529-1.535 |
| Steroid only | 27/47(57.4) | 12/22(54.5) | 0.821 | 0.889 | 0.321-2.463 |
| Steroid +IVIG | 13/47(27.7) | 6/22(27.3) | 0.973 | 0.981 | 0.315-3.052 |
| Add immunosuppressive | 4/47(8.5) | 4/22(18.2) | 0.252 | 2.389 | 0.538-10.612 |
| Steroid more than 5 weeks | 43/47(91.5) | 16/22(72.7) | **0.049** | 0.248 | 0.062-0.995 |
| Isolated ON | 6/47(12.8) | 5/22(22.7) | 0.298 | 2.01 | 0.540-7.483 |
| Isolated TM | 10/47(21.3) | 1/22(4.5) | 0.109 | 0.176 | 0.021-1.474 |
| Isolated ADEM | 15/47(31.9) | 8/22(36.4) | 0.715 | 1.219 | 0.421-3.530 |
| Isolated CE | 3/47(6.4) | 1/22(4.5) | 0.762 | 0.698 | 0.068-7.122 |
| Isolated BS | 3/47(6.4) | 0/22(0) | 0.999 | 0 | 0- |
| Mixed | 10/47(21.3) | 7/22(31.8) | 0.346 | 1.727 | 0.554-5.383 |

**Supplementary Table 2.2** Multivariate Analysis of Factors Associated with a Relapsing Course

| Variables | B | S.E. | Wald χ^2^ | P | OR | 95%CI |
| --- | --- | --- | --- | --- | --- | --- |
| Visual impairment | 1.439 | 0.626 | 5.281 | **0.022** | 4.215 | 1.236-14.377 |
| Headache | 1.166 | 0.601 | 3.767 | 0.052 | 3.209 | 0.989-10.419 |
| Steroid more than 5 weeks | -1.558 | 0.808 | 3.719 | 0.054 | 0.211 | 0.043-1.026 |

**Supplementary Table 3.1** Univariate Analysis of Factors Associated with Unfavourable Disability Outcome

| Variables | Favorable prognosis | Unfavourable prognosis | P | OR | 95%CI |
| --- | --- | --- | --- | --- | --- |
| Female | 26/56(46.4) | 2/13(15.4) | 0.055 | 0.21 | 0.043-1.034 |
| Age of onset | 16.5(7,31.75) | 26(7.5,57.5) | 0.058 | 1.03 | 0.999-1.062 |
| Fever | 25/56(44.6) | 5/13(38.5) | 0.686 | 0.775 | 0.225-2.666 |
| Visual impairment | 23/56(41.1) | 7/12(58.3) | 0.28 | 2.009 | 0.567-7.118 |
| Ocular movement pain | 12/56(21.4) | 2/12(16.7) | 0.712 | 0.733 | 0.141-3.807 |
| Visual field defects | 9/51(17.6) | 2/9(22.2) | 0.744 | 1.333 | 0.237-7.510 |
| Headache | 22/56(39.3) | 7/13(53.8) | 0.342 | 1.803 | 0.535-6.078 |
| Dizziness | 9/56(16.1) | 3/13(23.1) | 0.551 | 1.567 | 0.359-6.842 |
| Somnipathy | 9/56(16.1) | 2/13(15.4) | 0.951 | 0.949 | 0.179-5.028 |
| Nausea/vomiting | 6/56(10.7) | 5/13(38.5) | **0.021** | 5.208 | 1.282-21.163 |
| Bowel/bladder disturbance | 14/56(25.0) | 5/13(38.5) | 0.332 | 1.875 | 0.526-6.680 |
| Paresthesia | 19/56(33.9) | 3/13(23.1) | 0.453 | 0.584 | 0.144-2.378 |
| Limb weakness | 26/56(46.4) | 5/13(38.5) | 0.604 | 0.721 | 0.210-2.479 |
| Numbness | 17/56(30.4) | 2/13(15.4) | 0.287 | 0.417 | 0.083-2.088 |
| Seizure | 13/56(23.2) | 2/13(15.4) | 0.541 | 0.601 | 0.118-3.067 |
| Dysmnesia | 3/56(5.4) | 1/13(7.7) | 0.747 | 1.472 | 0.141-15.411 |
| Consciousness disorders | 14/56(25.0) | 2/13(15.4) | 0.464 | 0.545 | 0.108-2.766 |
| Speech disorders | 5/56(8.9) | 1/13(7.7) | 0.887 | 0.85 | 0.091-7.962 |
| CSF cell number（10^6/L） | 32(7.5,83.5) | 41(7.5,61) | 0.434 | 0.995 | 0.984-1.007 |
| CSF glucose (mmol/L) | 3.14(2.72,3.70) | 3.46±0.22 | 0.637 | 1.193 | 0.574-2.479 |
| CSF protein (mg/L) | 319(257.43,407.30) | 372.2(295,549.6) | 0.228 | 1.001 | 0.999-1.004 |
| CSF immunoglobulin IgG(mg/L) | 30.3(23.8,44.4) | 37.0(25.9,70.1) | 0.565 | 1.005 | 0.987-1.024 |
| CSF albumin (mg/L) | 196.1(143.69,278.0) | 214(157,379.5) | 0.206 | 1.002 | 0.999-1.005 |
| QIgG | 3.13(2.19,4.07) | 2.51(1.60,6.44) | 0.326 | 1.059 | 0.945-1.187 |
| QAlb | 4.47(3.4,6.68) | 4.78(4.34,9.86) | 0.235 | 1.086 | 0.948-1.243 |
| IgG Index | 0.70±0.04 | 0.75±0.09 | 0.581 | 2.073 | 0.156-27.605 |
| 24h intrathecal IgG synthesis rate | 1.04(0,5.03) | 1.86(0,7.43) | 0.442 | 1.012 | 0.982-1.043 |
| Type I isoelectric focusing pattern | 32/43(74.4) | 4/11(36.4) | **0.023** | 0.196 | 0.048-0.802 |
| Type Ⅱ isoelectric focusing pattern | 8/43(18.6) | 4/11(36.4) | 0.215 | 2.5 | 0.587-10.645 |
| Type Ⅳ isoelectric focusing pattern | 3/43(7.0) | 3/11(27.3) | 0.075 | 5 | 0.851-29.39 |
| Damage of BBB | 16/43(37.2) | 5/11(45.5) | 0.618 | 1.406 | 0.369-5.361 |
| EDSS at discharge | 1(0,2) | 3.27±0.63 | **0.005** | 1.529 | 1.138-2.055 |
| Total number of episodes | 1(1,2) | 2(1,4) | **0.009** | 2.007 | 1.188-3.393 |
| Steroid only | 32/56(57.1) | 7/13(53.8) | 0.829 | 0.875 | 0.260-2.940 |
| Steroid +IVIG | 16/56(28.6) | 3/13(23.1) | 0.69 | 0.75 | 0.182-3.086 |
| Add immunosuppressive | 7/56(12.5) | 1/13(7.7) | 0.629 | 0.583 | 0.065-5.203 |
| Steroid more than 5 weeks | 52/56(92.9) | 7/13(53.8) | **0.002** | 0.09 | 0.02-0.399 |

**Supplementary Table 3.2** Multivariate Analysis of Factors Associated with unfavourable disability outcome

| Variables | B | S.E. | Wald χ^2^ | P | OR | 95%CI |
| --- | --- | --- | --- | --- | --- | --- |
| Nausea/vomiting | 3.206 | 1.920 | 2.789 | 0.095 | 24.684 | 0.573-1063.04 |
| Type I electrophoresis | -5.510 | 2.347 | 5.513 | **0.019** | 0.004 | 0.000-0.402 |
| EDSS at discharge | 1.619 | 0.704 | 5.289 | **0.021** | 5.050 | 1.270-20.074 |
| Total number of episodes | 2.223 | 0.981 | 5.140 | **0.023** | 9.235 | 1.352-63.105 |
| Steroid more than 5 weeks | -6.619 | 2.826 | 5.486 | **0.019** | 0.001 | 0.000-0.339 |
